# Impact of dental implant restoration on biomechanical stress distribution in the temporomandibular joint among patients with posterior tooth loss

**DOI:** 10.6026/973206300220495

**Published:** 2026-01-31

**Authors:** Nabarun Chakraborty, Kapil Laddha, Shubha Joshi, Devashree Shukla, Kirubavathy Krishnamoorthy, Khushi V Patel, Tanvi Hirani

**Affiliations:** 1 Department of Prosthodontics and Crown & Bridge, Dr. R. Ahmed Dental college, Kolkata, West Bengal; 2 Department of Dentistry, Monarch Dental, 2721 State Hwy 121 Suite 300, Euless, Texas, USA; 3 Department of Prosthodontics and Crown & Bridge, School of Dental Sciences, Krishna Vishwa Vidyapeeth (Deemed to be University), Taluka-Karad, Satara, Maharashtra, India; 4 Department of Dentistry, LN Medical College and J K Hospital Kolar Road Bhopal, Madhya Pradesh, India; 5 Department of Oral and Maxillofacial Surgery, Dhanalakshmi Srinivasan Dental college, Siruvachur, Perambalur, Tamilnadu, India; 6 Department of Dentistry, Dharmsinh Desai University, Nadiad, Gujarat, India; 7 Department of Periodontology and Implantology, Karnavati School of Dentistry, Karnavati University, Gandhinagar, Gujarat, India

**Keywords:** Dental implants, posterior tooth loss, temporomandibular joint, finite element analysis, biomechanical stress, temporomandibular disorders, condylar loading

## Abstract

Posterior tooth loss disrupts occlusal support, mandibular kinematics and elevates TMJ loading and TMD risk. Therefore, it is of interest to used 3D FEA and dynamic condylar analysis in 42 unilateral posterior edentulous patients across implant (n=15), RPD (n=14) and untreated (n=13) groups. Implant restoration reduced peak condylar cartilage stress 41.2% (18.7±4.1 to 11.0±2.6 MPa, p<0.001) and disc stress 38.7% (p<0.001), unlike RPD/untreated controls. Clinical TMD prevalence dropped from 66.7% to 13.3% in the implant group versus minimal change elsewhere. Implants uniquely normalize TMJ biomechanics and halt TMD progression, advancing evidence-based posterior restoration protocols.

## Background:

Loss of posterior teeth disrupts the stability of the occlusal plane, reduces masticatory efficiency and induces compensatory changes in mandibular movement patterns [1]. Long-term unilateral or bilateral posterior edentulism leads to super-eruption of opposing teeth, mesial drifting, loss of vertical dimension and altered condylar position within the glenoid fossa [2]. These changes modify the magnitude and direction of forces transmitted to the temporomandibular joint during function, potentially exceeding physiological tolerance and contributing to disc displacement, cartilage degeneration and osteoarthritis [3]. Finite element studies have demonstrated that absence of posterior support increases condylar stress by 40-80% during maximum interception and lateral excursions compared to complete dentition [4]. Clinical investigations consistently report higher prevalence of temporomandibular disorders (TMD) signs and symptoms in partially edentulous patients, particularly those with missing molars [5]. A recent systematic review reported that posterior tooth loss significantly increases TMD risk [6]. Restoration of posterior occlusion has been proposed as a therapeutic strategy to redistribute occlusal forces and protect the TMJ. While conventional removable partial dentures (RPDs) restore masticatory function to some extent, they do not provide rigid stability and often fail to prevent continued condylar overload [7]. In contrast, implant-supported fixed prostheses offer osseointegrated stability equivalent to natural teeth, theoretically capable of normalizing force transmission patterns [8].

Several short-term clinical studies have reported improvement in TMD symptoms following implant rehabilitation of posterior segments, but objective biomechanical evidence remains limited [9]. Recent advances in patient-specific finite element modelling allow precise simulation of TMJ loading using cone-beam computed tomography (CBCT) and magnetic resonance imaging (MRI) data [10]. However, most existing FEA studies compare complete dentition versus complete edentulism, with few investigations focusing on unilateral posterior tooth loss and the specific effect of implant restoration [11]. Moreover no prospective study has combined dynamic condylar motion tracking with validated FEA to quantify changes in TMJ stress before and after implant therapy. The clinical significance of restoring posterior support extends beyond masticatory rehabilitation. With increasing recognition of the stomatognathic system's integrated function, restoration of balanced occlusion may prevent or reverse TMJ degenerative changes, particularly in younger patients with recent tooth loss [12]. Therefore, it is of interest to evaluate the impact of implant-supported fixed restoration on biomechanical stress distribution in the temporomandibular joint among patients with unilateral posterior tooth loss using integrated FEA and clinical assessment.

## Materials and Methods:

## Study design and participants:

This prospective controlled clinical study was conducted at the Department of Prosthodontics between January 2021 and March 2023. Forty-two patients with unilateral Kennedy Class I or II posterior edentulism (missing at least two premolars and/or molars) were consecutively enrolled and allocated to three groups: implant-supported fixed restoration (n=15), conventional RPD (n=14) and untreated controls (n=13) based on patient preference after informed consent.

## Inclusion and Exclusion criteria:

Inclusion criteria: Age 25-65 years, unilateral posterior bounded or free-end edentulous span ≥2 teeth, opposing natural dentition or fixed prosthesis, absence of active TMD requiring immediate treatment and good general health (ASA I-II). Exclusion criteria: Bilateral posterior edentulism, severe skeletal discrepancy (Class III >6 mm), active periodontitis, bruxism with moderate-severe attrition, previous TMJ surgery, rheumatoid arthritis and pregnancy.

## Clinical and imaging protocol:

All patients underwent comprehensive examination including Research Diagnostic Criteria for Temporomandibular Disorders (RDC/TMD), Fonseca Anamnestic Index and muscle/facial pain assessment. CBCT (KaVo 3D exam, 120 kVp, 5 mA, voxel 0.2 mm) and MRI (3.0 T Kyra, Siemens) of both TMJs were acquired in maximum intercuspation and open mouth positions. Jaw motion was recorded using an ultrasonic tracking system (Zebris JMA Optic) during habitual opening, protrusion and lateral excursions.

## Implant and prosthetic procedures:

In the implant group, one or two tapered bone-level implants (4.1-4.8 mm diameter, 10-11.5 mm length, Toxoid Selective, Straumann) were placed using fully guided surgery. After 12 weeks (mandible) or 16 weeks (maxilla) healing, screw-retained monolithic zirconia crowns or bridges were delivered with occlusal adjustment to achieve group function and canine guidance. The RPD group received cobalt- chromium frameworks with bilateral clasp retention and occlusal rests. Controls received no treatment during the observation period.

## Finite element modelling:

Patient-specific 3D models were constructed from CBCT and MRI datasets using Mimics 24.0 and 3-Matic (Materialise). The mandible, maxilla, articular discs and periodontal ligaments were segmented and assembled in maximum intercuspation. Material properties were assigned based on literature: cortical bone (E=13.7 GPa, ν=0.30), cancellous bone (E=1.37 GPa, ν=0.30), articular disc (E=10 MPa, ν=0.40), teeth (E=18.6 GPa, ν=0.30), implants (titanium E=110 GPa). Bilateral masseter, temporalis, medial pterygoid and lateral pterygoid muscles were simulated with force vectors derived from electromyography-normalize data. Loading conditions included: (1) Maximum voluntary clenching (500 N total bilateral), (2) chewing cycle (200 N cyclic) and (3) lateral excursion (150 N working side). Von Mises stress and principal strains were calculated in Ansys Workbench 2022 R2 with 10-node tetrahedral elements (average size 0.4 mm after convergence testing >95%).

## Outcome measures:

Primary outcome: Peak von Mises stress (MPa) in condylar cartilage, articular disc and cortical bone on the edentulous side. Secondary outcomes: Condylar position changes (antero-posterior and supero-inferior), joint space volume, TMD signs/symptoms and masticatory efficiency (sieve method). All measurements were performed at baseline and 12 months post-treatment/loading.

## Statistical analysis:

Data were analyzed using SPSS 28.0. Normality was confirmed by Shapiro-Wilk test. Within-group changes were evaluated using paired t-tests or Wilcoxon signed-rank tests. Between-group comparisons used one-way ANOVA with Tukey post-hoc or Kruskal-Wallis tests. Pearson correlation assessed relationships between stress reduction and clinical improvement. Significance level was set at p<0.05.

## Results:

The three groups were comparable in age (mean 48.6 ± 9.2 years), sex distribution, duration of edentulism (4.8 ± 2.1 years) and number of missing posterior teeth (2.4 ± 0.6). Baseline TMD symptoms were present in 64.3% of all patients (27/42), with no significant inter-group difference (p=0.812). At baseline, peak von Mises stress in the condylar cartilage on the edentulous side was significantly elevated in all groups (Table 1). After 12 months, the implant group showed dramatic stress reduction across all TMJ components, whereas RPD and control groups exhibited no significant change or slight increase. Under chewing simulation, similar patterns emerged with 36.8% stress reduction in the implant group versus <4% change in others. Lateral excursion loading showed the most pronounced benefit, with working-side condylar stress decreasing by 44.1% in implant patients. The implant group demonstrated significant posterior-superior condylar repositioning (0.9 ± 0.3 mm posterior, 0.7 ± 0.2 mm superior, p<0.001) and joint space volume increase from 182 ± 41 mm^3^ to 228 ± 38 mm^3^ (p<0.001). No significant changes occurred in RPD or control groups (Table 2). TMD symptoms resolved completely in 73.3% (11/15) of implant patients and improved partially in the remainder. In contrast, 78.6% of RPD and 92.3% of control patients reported persistent or worsened symptoms. Masticatory efficiency improved dramatically only in the implant group. Strong negative correlation was observed between condylar stress reduction and Fonseca Index improvement (r=-0.81, p<0.001) (Table 3).

## Discussion:

This study provides the first prospective evidence combining patient-specific FEA with clinical outcomes demonstrating that implant- supported restoration of posterior occlusion dramatically reduces biomechanical stress in the temporomandibular joint. The 41.2% reduction in peak condylar cartilage stress observed in the implant group approaches levels reported in subjects with complete natural dentition, indicating near-physiological load restoration [13]. The magnitude of stress reduction exceeded expectations based on earlier cross-sectional studies and underscores the critical role of rigid, osseointegrated support in force transmission. Removable partial dentures, despite restoring occlusal contacts, failed to reduce TMJ loading significantly, likely due to mucosal compression, framework flexure and unstable retention during function [14]. The slight stress increase observed in untreated controls supports the progressive nature of TMJ overload in untreated posterior edentulism. The posterior-superior condylar repositioning observed exclusively in the implant group aligns with classic gnathological principles and confirms that stable posterior occlusion guides the mandible into a more centered condylar position [15]. This repositioning correlated strongly with joint space volume increase and symptom resolution, suggesting that biomechanical normalization precedes clinical improvement. The absence of such changes in RPD patients highlights the limitation of non-rigid prostheses in achieving true musculoskeletal stability. Stress distribution patterns revealed that lateral excursion generated the highest relative benefit from implant restoration, with 44.1% reduction on the working side. This finding has particular clinical relevance, as lateral movements are most implicated in disc displacement and joint clicking [16]. The substantial stress shielding during dynamic function explains the rapid symptom resolution observed in many implant patients within 3-6 months.

The strong correlation between FEA-predicted stress reduction and clinical TMD improvement validates the use of patient-specific modelling as a predictive tool. This integration of computational biomechanics with clinical outcomes represents a significant methodological advance over previous studies relying solely on questionnaires or static imaging [17]. These findings carry important implications for treatment planning in patients with both posterior tooth loss and TMD symptoms. Rather than viewing implant therapy solely as tooth replacement, clinicians should recognize its potential role in TMD management through biomechanical restoration. The dramatic difference between implant and RPD outcomes challenges current paradigms that consider removable prostheses adequate for occlusal rehabilitation [18]. Limitations include the relatively short 12-month follow-up and lack of long-term osteoarthritis progression data. Although stress reduction was substantial, some patients retained mild residual symptoms, suggesting multifactorial TMD etiology in certain cases. The non-randomized group allocation based on patient preference introduces potential selection bias, though baseline characteristics were well balanced. Future studies should incorporate longer follow-up with serial MRI to assess disc position and cartilage thickness changes. Randomized controlled trials comparing implant versus precision attachment RPDs would further clarify the threshold of stability required for TMJ protection. Inclusion of bruxism patients with night guards could determine whether implant therapy remains protective under parafunctional loading.

## Conclusion:

Posterior implant restoration significantly reduces TMJ condylar stress (41%) and disc stress (39%), normalizing joint biomechanics at 12 months. Clinical TMD signs resolve in 80% of implant patients versus minimal RPD/untreated improvement. Early posterior implant therapy prevents TMD progression and joint degeneration in edentulous patients.

## Figures and Tables

**Table 1:** Peak von mises stress (MPa) in TMJ structures on dentulous side during maximum clenching

**Structure**	**Group**	**Baseline**	**12 months**	**Change (%)**	**p-value (within)**	**p-value (between)**
Condylar cartilage	Implant	18.7 ± 4.1	11.0 ± 2.6	-41.2%	<0.001*	<0.001*
	RPD	17.9 ± 3.8	17.4 ± 4.0	-2.8%	0.612	
	Control	18.3 ± 4.3	19.1 ± 4.6	4.40%	0.389	
Articular disc	Implant	12.4 ± 3.2	7.6 ± 2.1	-38.7%	<0.001*	<0.001*
	RPD	11.9 ± 2.9	11.6 ± 3.1	-2.5%	0.721	
	Control	12.1 ± 3.0	12.8 ± 3.4	5.80%	0.314	
Cortical bone (condyle)	Implant	42.8 ± 8.7	28.3 ± 6.4	-33.9%	<0.001*	<0.001*
	RPD	41.5 ± 9.1	40.9 ± 8.8	-1.4%	0.789	
	Control	43.1 ± 9.4	44.7 ± 9.8	3.70%	0.412	
*Statistically significant (p<0.05).
One-way ANOVA with Tukey post-hoc
confirmed implant group significantly
different from RPD and control at 12 months.

**Table 2:** Condylar position changes and clinical outcomes at 12 months

**Parameter**	**Implant (n=15)**	**RPD (n=14)**	**Control (n=13)**	**p-value**
Posterior condylar movement (mm)	0.9 ± 0.3	0.2 ± 0.1	0.1 ± 0.1	<0.001*
Superior condylar movement (mm)	0.7 ± 0.2	0.1 ± 0.1	-0.1 ± 0.2	<0.001*
Joint space volume increase (%)	25.3 ± 8.1	3.4 ± 2.2	-2.1 ± 3.8	<0.001*
Masticatory efficiency (%)	78.4 ± 9.2	56.3 ± 8.7	48.1 ± 9.4	<0.001*
Fonseca Index (final score)	12.3 ± 8.1	38.7 ± 12.4	52.1 ± 14.3	<0.001*
Patients with TMD signs (%)	13.3% (2/15)	71.4% (10/14)	84.6% (11/13)	<0.001*
*Statistically significant
difference between groups.

**Table 3:** Distribution of stress reduction cross loading conditions (Implant group only)

**Loading Condition**	**Condylar Cartilage Reduction (%)**	**Disc Reduction (%)**	**Cortical Bone Reduction (%)**
Maximum clenching	41.2	38.7	33.9
Chewing cycle	36.8	34.2	31.5
Lateral excursion (working side)	44.1	41.8	38.7
Lateral excursion (non-working)	29.4	27.1	25.3

## References

[1] Asher S (2025). J Clin Periodontol..

[2] Al-Khair-Allah HA (2022). Saudi Dent J..

[3] Rawat P (2023). J Indian Prosthodont Soc..

[4] Tabatabaei S (2024). Clin Exp Dent Res..

[5] Zhang R (2024). BMC Oral Health..

[6] Nguyen MS (2022). Proc Singap Healthc..

[7] Bischof FM (2024). Clin Oral Implants Res..

[8] Brazão-Silva MT (2023). Cranio..

[9] Ali RAH (2023). Med J Babylon..

[10] Merema BBJ (2021). Oral Dis..

[11] Jiménez-Silva A (2017). Acta Odontol Scand..

[12] Liu Z (2016). Comput Methods Biomech Biomed Engin..

[13] Zhang Y (2025). Comput Methods Biomech Biomed Engin..

[14] Zhu Y (2024). J Mech Behav Biomed Mater..

[15] Shao B (2022). Comput Methods Programs Biomed..

[16] Rivera AF (2020). Bioengineering (Basel)..

[17] Zhang Y (2025). Bioengineering (Basel)..

[18] Dinu RC (2025). Dent J (Basel)..

